# Multitask Learning and GCN-Based Taxi Demand Prediction for a Traffic Road Network

**DOI:** 10.3390/s20133776

**Published:** 2020-07-05

**Authors:** Zhe Chen, Bin Zhao, Yuehan Wang, Zongtao Duan, Xin Zhao

**Affiliations:** School of Information Engineering, Chang’an University, Xi’an 710064, China; zchen@chd.edu.cn (Z.C.); bzhao@chd.edu.cn (B.Z.); yhwang@chd.edu.cn (Y.W.); xinzhao@chd.edu.cn (X.Z.)

**Keywords:** taxi demand prediction, graph neural network, GPS trajectory of taxis, spatial-temporal model, deep learning

## Abstract

The accurate forecasting of urban taxi demands, which is a hot topic in intelligent transportation research, is challenging due to the complicated spatial-temporal dependencies, the dynamic nature, and the uncertainty of traffic. To make full use of the global and local correlations between traffic flows on road sections, this paper presents a deep learning model based on a graph convolutional network, long short-term memory (LSTM), and multitask learning. First, an undirected graph model was formed by considering the spatial pattern distribution of taxi trips on road networks. Then, LSTMs were used to extract the temporal features of traffic flows. Finally, the model was trained using a multitask learning strategy to improve the model’s generalizability. In the experiments, the efficiency and accuracy were verified with real-world taxi trajectory data. The experimental results showed that the model could effectively forecast the short-term taxi demands on the traffic network level and outperform state-of-the-art traffic prediction methods.

## 1. Introduction

Intelligent transportation systems (ITS) are one of the most important directions in the development of future transportation systems. ITS effectively integrate advanced information and communication technologies to form a real-time, accurate, and efficient transportation management system. As part of ITS, traffic prediction is crucial, with the goal of predicting short- or long-term traffic volumes based on historical traffic data (such as traffic flow, vehicle speed, etc.) and the state of road networks.

Taxis play an important role in urban public transportation, but because of the uncertainty and dynamic nature of taxi demand, it is more challenging to tell where and when a vacant taxi could find a passenger. In addition to online car hailing, there are still a tremendous number of taxis collecting passengers in the traditional way, that is, cruising on urban roads to find passengers. The information gap between taxis and passengers is the main reason for the imbalance in supply and demand. For example, when there is a shortage of taxis in certain areas, there could be an excessive supply in other areas. Therefore, it is crucial to forecast where, when, and how many potential passengers are hailing taxis. By analyzing and predicting the needs of taxi passengers, the dynamic changes of travel demand in different areas of the city can be more accurately reflected. This not only helps travelers choose time- or cost-saving travel routes, but also helps city managers to carry out informed traffic planning and public vehicle resource scheduling, thereby alleviating traffic congestion and reducing the waste of public resources [1]. As complex temporal-spatial dependencies are inherent in the dynamics of traffic, it is difficult to achieve an accurate and efficient prediction [2]. Therefore, this area of interest has attracted remarkable research attention in recent decades.

According to the length of the time period, traffic prediction problems can be generally divided into two categories: short-term prediction (5–30 min) and medium- to long-term prediction (more than 30 min) [3]. Traditional traffic prediction methods include parametric methods and non-parametric methods. The former methods are model driven, including time series models such as Auto-regressive integrated moving average (ARIMA) and its variants [4], Kalman filtering [5], and so forth. Such methods have strict assumptions about data characteristics. For example, ARIMA assumes that the time series are stationary, even though an actual traffic flow sequence rarely satisfies this condition. Therefore, such methods cannot effectively extract complex nonlinear spatial and temporal relations. With the widespread use of GPS equipment, mobile communication devices, and so on, a large amount of traffic data are generated every day, which has promoted the emergence of data-driven methods. Non-parametric methods are typical data-driven methods, including those based on traditional machine learning and deep learning. Traditional machine learning methods include k-nearest neighbor (KNN), support vector regression (SVR), artificial neural networks, and so the like [6]. The prediction accuracies of these methods are generally better than those of the model-driven methods. However, as their prediction performance mainly depends on handcraft features engineering, the nonlinear spatio-temporal relationship of traffic data cannot be effectively extracted, and high-dimensional data cannot be processed. In the deep learning model, the stacked autoencoder (SAE) and deep belief network (DBN) cannot easily extract the temporal and spatial correlations of traffic data simultaneously, and they almost completely ignore the spatial characteristics, so the expression ability is rather limited. A convolutional neural network (CNN) is particularly suitable for processing regular data such as two-dimensional grids, and it can effectively extract spatial correlations, while recurrent neural networks (RNNs) are suitable for extracting temporal correlations. Therefore, a combination of a CNN and RNNs can effectively extract spatial and temporal correlations. However, when using a CNN to extract the spatial correlations of traffic flow data, it is necessary to divide the traffic network into two-dimensional regular grids, which destroys the original structure of the traffic network, and the ability to extract spatial correlations is still insufficient.

The graph convolutional neural network (GCN) has emerged in recent years. It is particularly suitable for extracting the spatial correlation of irregular, non-Euclidean graph data, which makes it an effective tool for extracting the spatial features of traffic flow. The GCN has achieved good results in predicting traffic speed [7]. Generally, in such tasks, road sections or traffic observation points are taken as nodes, and edges are generated according to the connectivity of road sections or observation points. There is a certain propagation effect of vehicle speed in adjacent road sections, therefore this kind of graph is conducive to fully mining the propagation mode of vehicle speed [8]. However, to predict travel flow, it is not appropriate to construct a graph that is based only on the connectivity of road sections. In an urban road network, not only are the taxi trips in adjacent sections correlated, but the non-adjacent road sections with the same function also have similar travel flow patterns [9] and certain correlations. Therefore, it is necessary to consider local and global correlations when constructing the graph. In addition to these machine-learning-based traffic prediction methods, there are also some methods that are not supervised (e.g., running a real-time tracking system through traffic cameras). These methods can tell when and how many taxis go through a surveillance area, but they fail to tell where, when, and how many passengers need cabs, and it is difficult to predict citywide traffic.

## 2. Related Works

A large number of methods based on deep neural networks have been applied to traffic prediction problems, such as Origin-Destination (OD) flow prediction, vehicle demand prediction, and so forth. Yao et al. [10] proposed a Deep Multi-View Spatial-Temporal Network (DMVST-Net), that used CNN and LSTM to extract local spatial features, semantic features, and temporal features to predict taxi demand. Liu et al. [11] proposed a contextualized spatial-temporal network (CSTN) based on ConvLSTM and CNN to predict taxi OD flow using similar features. The network consisted of three parts: local spatial context (LSC), temporal evolution context (TEC), and global correlation context (GCC), which extracted local spatial features, temporal features, and global spatial features, respectively. Duan et al. [12] proposed a hybrid deep network model based on ConvLSTM, Conv2DTranspose, and SeparableConv2D to predict taxi OD flows. In the model training, they took into account the correlation between travel time and OD flows to improve prediction accuracy.

The GCN has the ability to effectively extract complex spatial correlations, justifying its ability to process highly nonlinear data in a non-Euclidean space. In the very beginning, when GCN was used in traffic prediction tasks, intersections were usually used as nodes and road sections between intersections were used as edges. In this way, Wu et al. [13] proposed the GAT-LSTM module with an integrated attention mechanism in the process of graphing input-to-state and state-to-state transitions, and they built an end-to-end coding prediction network based on the GAT-LSTM module that could simultaneously predict the traffic flows of multiple roads. Wang et al. [14] adopted a new scheme to construct graphs that took observation positions as nodes and the transition values of travel time functions as the weights of edges, for which a traffic flow graph and an adjacency matrix were constructed according to the time slots. In addition, a dual-flow network was constructed, including the predicted traffic flow and graph prediction flow. In traffic flow prediction, the GCN was used to extract spatial features and 3D convolution was used to extract temporal features. Graph prediction flow was achieved by stacking multiple layers of the CNN, which could capture the spatially dependent changes in the traffic data. By employing two short-term prediction tasks to carry out two-step predictions, the accuracy of the long-term flow prediction was improved. This work could achieve the dynamic prediction of the graph structure, and it could effectively extract the spatially dependent changes in the prediction task. Subway passenger flow data also have the characteristics of high nonlinearity and complicated spatial-temporal relationships. Therefore, predicting subway passenger flow is also difficult when extracting the complex spatial-temporal dependencies. In constructing graphs based on subway stations in urban areas, careful use of the graph-adjacent rectangle construction strategy and map feature matrix or the use of a graph convolutional neural network could both effectively solve this problem. Li et al. [15] predicted urban subway flow. First, they divided an urban road network into grids to ensure that each station was in a grid. Taking each grid as a node and the average daily travel times between nodes as the weight of the edges, they constructed the inflow and outflow matrices in time slots. Then, a CNN was used to extract the spatio-temporal correlations of subway traffic to predict subway traffic flow. The flow prediction of a bike sharing system could also be processed from the use of a graph. Chai et al. [16] proposed a multigraph convolutional neural network to predict site-level bicycle sharing traffic flows. Specifically, they constructed multiple graphs in a shared bicycle system to reflect the heterogeneous inter-station relationships. Then, the graphs were combined and a graph convolution network was used to predict the future station-level bicycle flow. Its key goal was to observe the bicycle sharing system from the perspective of a graph. Traditional travel time estimation methods generally rely on given route information, which cannot be used to estimate real-time dynamics. To estimate the travel time in real time, Li et al. [17] put forward a new multitask representation learning model to estimate travel time. Based on the road network structure and the hidden information in the temporal dimension, a new spatial and temporal map representation was established. It could fully mine the spatial and temporal dimension features of travel time; introduce historical path information during model training, such as the number of road sections, vehicle travel distance, and other information; conduct multitask learning; and predict travel time, travel distance, road section numbers, and other information at the same time. While the model could perform related tasks, the model achieved good results in the main task of estimating travel time. Zheng et al. [18] designed a multilayer attention graph neural network (GMAN) to forecast traffic flow. It was implemented based on a new spatio-temporal gating fusion attention mechanism and a shift attention mechanism, which effectively improved the performance of the long-term traffic flow prediction. Yao et al. [19] proposed a novel spatial-temporal dynamic network (STDN), in which a flow gating mechanism was introduced in order to obtain similar dynamics between locations and a periodically shifted attention mechanism was designed to handle long-term periodic temporal shifting, which forecasted regional level taxi demand.

## 3. Motivation

**Figure 1:**
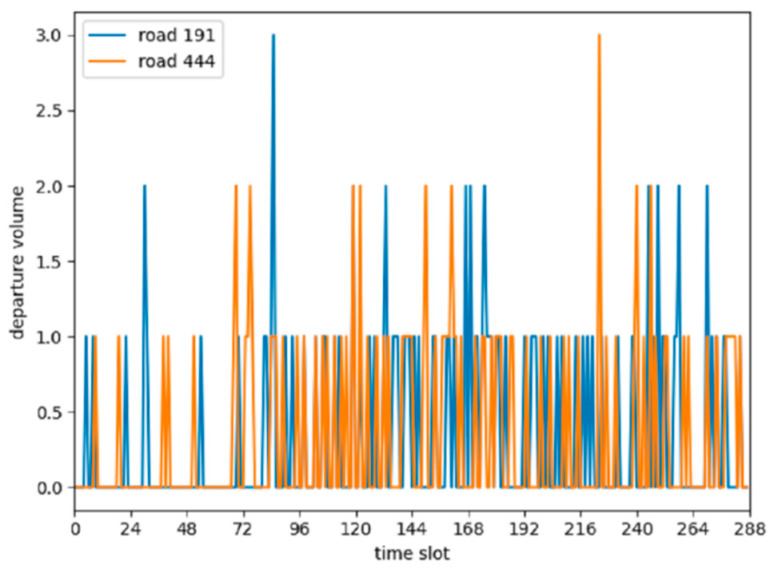
Similar traffic flow patterns on road 191 and road 444.

**Figure 2:**
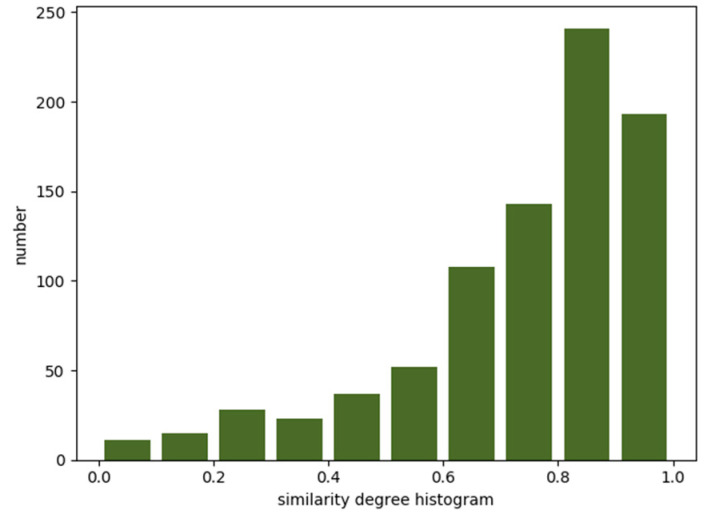
The correlation of the departure flow and arrival flow sequences.

For the existing GCN-based [20] traffic volume prediction methods, graphs are directly constructed according to the topology of the road networks. This type of construction is more suitable to the prediction of traffic speed because the speed of vehicles on adjacent roads is usually highly correlated [8]. In an urban road network, the disjointed road sections with similar traffic functions such as main roads near transportation junctions or business zones usually have similar traffic flow patterns [9]. As shown in Figure 1, we divided a day into 288 time slots, with each time slot taking 5 min. Figure 1 shows two sequences of taxi departure flows for two functionally similar roads, road 191 and road 444 in the city of Xi’an. Both roads were located near schools, and these two schools had the same schedule. It can be seen that the roads had similar taxi demand patterns, and the similarity that was calculated according to Equation (2) was greater than 0.891. In addition, we found that the same road section showed certain similarities for departure and arrival flow patterns in different time slots, which meant that the prediction tasks for departure and arrival flows could be regarded as two correlated tasks. As shown in Figure 2, we divided the similarity range (interval [0,1]) into bins. The width of each bin was 0.1. The height of each bin indicated the number of road sections that had the same range of similarity, calculated according to Equation (2). *ε* was set as 0. As also shown in Figure 2, for most road sections, the similarity between the departure and arrival flows was greater than 0.6, which meant that to a certain extent, there was a correlation between the departure and arrival demands in most city road sections. This gave us an opportunity to incorporate multitask learning to jointly forecast departure and arrival flows. Through parameter sharing, multitask learning allows deep neural networks to learn more general features, which greatly reduces the risk of overfitting and enables the model to have better generality [21]. However, most existing methods do not take this correlation into account.

Inspired by this, we proposed a GCN and multitask learning-based model to take advantage of the different kinds of correlations existing in traffic networks. More specifically, we first divided each day into multiple time slots. Then, we counted the departures and arrivals of taxis in each road section for every time slot, and at the same time, we extracted the origin–destination matrix and we obtained the departure and arrival flow sequences at consecutive time slots for each road section. Next, we used the fast dynamic time warping algorithm [22] to calculate the distances of the departure or arrival flow sequences between different road sections on multiple days to find correlations behind them. By taking each road section as a node of the graph and the similarity between two connected nodes as an edge weight, the adjacency matrix was obtained. Then, two types of graphs based on the sequence similarity were constructed for each road section, that is, the similarity graph of the taxi departure flow and the similarity graph of taxi arrival flow, which were suitable for mining global spatial features. Further, by extracting the links in the original road network, the departure flow and arrival flow graphs were constructed according to the topology of the road networks, which were suitable for mining local spatial features. In addition to GCN, considering that the long short-term memory network (LSTM) incorporated a gated linear unit (GLU), which could not only effectively reduce gradient dispersion but also retain the non-linear capability of the network [23], we integrated LSTM into our model to extract temporal features in the taxi flow sequence.

In summary, the main contributions of our work include the following:

- A new GCN-based graph network was proposed to predict taxi departure and arrival flows. In order to achieve road-level predictions, instead of dividing the urban space into grids, road sections were used as nodes and taxi demand was used as a feature of a node in constructing the traffic flow graph.
- Two types of traffic flow graphs were constructed based on local and global correlations. These graphs were referred to as the direct and indirect graph. The direct graph was constructed based on the topology of the road network, and it integrated local connectivity and spatial correlation between road sections. In contrast, the indirect graph took the global correlations between all road sections in the traffic network into consideration whether they were connected or not.
- Two kinds of correlated tasks—that is, the departure flow prediction and the arrival flow prediction—were combined, and a multitask learning strategy was used to boost the learning process, avoid overfitting, and obtain a more generalized result.
- Comprehensive experiments were performed with real-world taxi trajectories, and the results showed that our proposed method was superior to the existing methods.

## 4. Preliminaries

In this section, we briefly introduce the taxi demand prediction problems and the related definitions.

*Road section*: The road section was defined as the road connecting two intersections.

*Taxi departure flow in a road section*: If the length of a time slot was t minutes, a total of 24×60∕t time slots were obtained for each day. For the time slot k, the number of times that passengers entered a taxi in road section r was called the taxi departure flow on road section r in time slot k, denoted as $X_{r}^{k}$.

*Taxi arrival flow in a road section*: If the length of a time slot was t minutes, a total of 24×60÷t time slots were obtained for each day. For time slot k, the number of times that passengers got out of a taxi in road section r was called the taxi arrival flow on road section r in time slot k, denoted as $H_{r}^{k}$.

*Taxi departure flow sequence in a road section*: The taxi departure flow of road section r for consecutive time slots formed a departure flow sequence, denoted as $X_{r}=\left\{X_{r}^{1},X_{r}^{2},\cdots ,X_{r}^{K}\right\}$, in which K was the number of time slots. If the total number of road sections was R, the taxi departure flow on R road sections within K time slots could be represented by the matrix $X\in {\mathbb{R}}^{K\times R}$.

*Taxi arrival flow sequence in a road section*: The taxi arrival flow of road section r for consecutive time slots formed an arrival flow sequence, denoted as $H_{r}=\left\{H_{r}^{1},H_{r}^{2},\cdots ,H_{r}^{K}\right\}$, in which K was the number of time slots. If the total number of road sections was R, the taxi arrival flow on R road sections within K time slots could be represented by the matrix $H\in {\mathbb{R}}^{K\times R}$.

*The similarity of the departure flow sequences for different road sections*: The similarity between the departure flow sequences of road sections i and j was denoted as $\omega_{i,j}$. The distance of two departure flow sequences $X_{i}$ and $X_{j}$, denoted as $L_{\left(X_{i},X_{j}\right)}$, could be calculated with the fast dynamic time warping (FAST-DTW) algorithm [22], for which the Euclidean distance is commonly used. The Euclidean distance between point $\left(x_{1},y_{1}\right)$ and point $\left(x_{2},y_{2}\right)$ was defined as shown in Equation (1). If the lengths of $X_{i}$ and $X_{j}$ were both K, we could construct a matrix $P\in {\mathbb{R}}^{K\times K}$, in which the element of position (i,j) was the Euclidean distance between point $X_{ii}$ and point $X_{jj}$. In the matrix $P\in {\mathbb{R}}^{K\times K}$, the optimal warp path from position (0,0) to position (K,K) was searched; this path had the smallest sum of elements. The distance $L_{\left(X_{i},X_{j}\right)}$ was the number of elements in the optimal warp path.

(1) $$d=\sqrt{{\left(x_{2}-x_{1}\right)}^{2}+{\left(y_{2}-y_{1}\right)}^{2}},$$

The similarity between the departure flow sequence of road sections i and j could be calculated with the following formula [3]:

(2) $$\omega_{i,j}=\left\{ \begin{array}{ll} e^{-\frac{L_{\left(X_{i},X_{j}\right)}^{2}}{\sigma^{2}}}, & i\neq j{\;\mathrm{and}\;e}^{-\frac{L_{\left(X_{i},X_{j}\right)}^{2}}{\sigma^{2}}}\geq \varepsilon \\ 0\;, & \;\mathrm{otherwise}. \end{array}\right.,$$

in which $\sigma^{2}$ and ε were used to control the sparsity of the adjacency matrix W. When $\sigma^{2}$ was fixed, the larger the ε, the sparser the matrix. Thus, the constructed graph was simpler, but the similarity between some pairs of actually similar road sections was ignored. This did not accurately represent the similarity between road sections. In contrast, the smaller the ε, the denser the matrix. Thus, the constructed graph was more complex and the computational complexity was higher. For this case, some road sections with low similarity were taken into account; this was not cost-effective. However, when ε was fixed, the smaller the $\sigma^{2}$, the sparser the matrix. We set ε as 0.5 and $\sigma^{2}$ as 1000 to avoid the matrix being too sparse. ε and $\sigma^{2}$ took the empirical value, and $\sigma^{2}$ was usually an integer power of 10.

*The similarity of the arrival flow sequences for different road sections:* The method that was used to calculate the similarity of the arrival flow sequences for different road sections was similar to that for the arrival flow sequences.

*Local relationship graph of a road section*: Graph G=(V,E,A) was defined, where V is the set of nodes and E is the set of edges. Each road section served as a node. $A\in {\mathbb{R}}^{\left|V\right|\times \left|V\right|}$ represented an adjacency matrix indicating the relationship between nodes, which was generated according to the connectivity of the road sections in the traffic road network. If road section i and road section j were directly connected, entry $a_{ij}$ of the adjacency matrix was 1; otherwise it was 0. The local relationship graph of the road sections could also be called a direct relationship graph of the road sections.

*Global relationship graph of the road sections*: In the global relationship graph of the road sections, each road section also served as a node, and the corresponding element in the adjacency matrix was calculated according to Equation (2); that is, $a_{ij}=\omega_{ij}$. A global relationship graph of the road sections could also be called an indirect relationship graph of the road sections.

*Taxi departure flow prediction for road sections*: For a certain road section, given the departure flow in a previous k time slot, i.e., $X^{1},X^{2},\cdots ,X^{k}$, the goal of the problem was to predict $X^{k+1}$.

## 5. The Proposed Method

In this section, the proposed taxi demand prediction model is introduced.

### 5.1. Overview of the Framework

**Figure 3:**
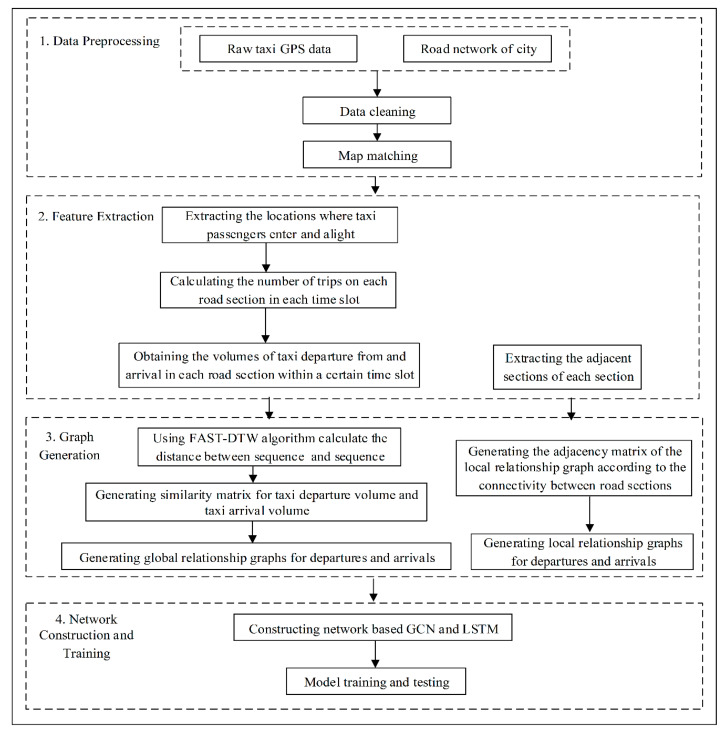
The overall framework of the proposed method.

The overall framework of the proposed method is shown in Figure 3. The method consisted of four parts, namely, data preprocessing, feature extraction, graph generation, and network construction and training. The data preprocessing included data cleaning and map matching. The purpose of the data cleaning was to remove duplicate and invalid records. The map matching was done to correct the collected GPS data to ensure that the data fell on the road section it belonged to. The map-matching algorithm based on Hidden Markov [24] was used, and we performed map matching on all of the trajectory data. Then, the locations where the taxi passengers entered and alighted could be extracted from the taxi trajectories, and the travel times on each road section were also calculated according to the time slot. Thus, the taxi departure and arrival flows in each road section were obtained within a certain time slot. However, for each road section, the adjacent road sections were extracted according to the topology of the urban road network. During the graph generation, four kinds of graphs were generated, including the global and local relationship graphs for departures and for arrivals. The adjacency matrix of the local relationship graph was generated according to the connectivity between road sections. In contrast, the adjacency matrix of the global relationship graph was generated by calculating the departure/arrival flow sequence similarity among different road sections. The purpose of the last part was to predict the departure flow based on GCN and LSTM networks, integrating a multitask learning strategy to train the model. In the multitask learning, the taxi departure flow prediction was taken as the main task, and the taxi arrival flow prediction was the related task, which was meant to help improve the prediction accuracy.

### 5.2. Deep Neural Networks

**Figure 4:**
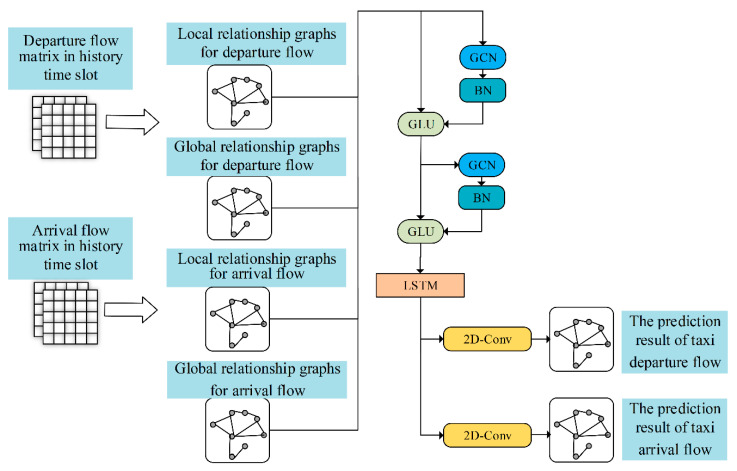
Multitask GCN-LSTM (MGLN) network.

The proposed multitask GCN-LSTM (MGLN) network is shown in Figure 4. The network consisted of three parts: the spatial feature extraction module (local spatial feature and global spatial feature), temporal feature extraction module, and feature fusion module. The spatial feature extraction module was produced by stacking two layers of GCN + GLU. The local spatial features—that is, the relationships between departure or arrival flows among adjacent road sections—could be extracted by processing the local relationship graph of the road sections. In the same way, the global spatial features describing the relationships of the departure or arrival flows between disjointed road sections could be extracted through processing the global relationship graph. The LSTM network was used to extract temporal features, which could be used to calculate the long-term dependence. The feature fusion module used two-dimensional convolution to integrate the prediction results from the local and global relationship graphs to obtain the final prediction.

#### 5.2.1. Local and Global Spatial Feature Extraction Module

In the module, the road sections were set as graph nodes whose features included historical departure or arrival flow sequences generated in the road sections represented by these nodes. Letting the total number of road sections be R, and the number of history time slots be K, then the feature matrix of the graph was $X\in {\mathbb{R}}^{K\times R}$, and the adjacent matrix was $A\in {\mathbb{R}}^{R\times R}$. In the module, two layers of GCN + GLU were used with a batch normalization layer added between the GCN and GLU. The behavior of the GCN could be represented as follows [25]:

(3) $$f(X,A)=Relu(\hat{A}XW),$$

where X is the feature matrix; $\hat{A}={\tilde{D}}^{-1/2}\tilde{A}{\tilde{D}}^{-1/2}$, $\tilde{A}=A+I$, and $\tilde{D}$ represents degree matrices, Relu() is a non-linear activation function, and W is the network parameter. Graph convolution methods are often categorized as spectral and non-spectral approaches. The GCN that we used was a spectral approach that was based on the spectral representation theory of the graphs.

After integrating the GLU and adding the BN layer, Equation (3) was changed to

(4) $$f(X,A)=BN\left(Relu(\hat{A}XW)\right)+\sigma \left(BN\left(Relu(\hat{A}XW)\right)\right),$$

where BN() is a batch normalization function and σ() is a non-linear activation function. In our method, a Leaky ReLU was used. The GCN, BN, and GLU were grouped together to form a function unit, and two of them were stacked to extract spatial features. The dimensions of the module output were K×R.

#### 5.2.2. Temporal Feature Extraction Module

**Figure 5:**
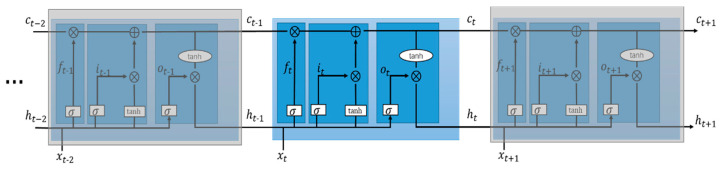
Network structure of LSTMs.

As is known, recurrent neural networks such as RNN and LSTM are capable of extracting temporal features from time sequences. Compared with RNN, LSTM can capture dependencies between long-term items and avoid the gradient-vanishing problem by importing a gate mechanism. In our model, a multilayer LSTM was used to extract the temporal features hiding in the traffic flow sequences. The architecture of the LSTM is shown in Figure 5. A basic LSTM unit was mainly composed of three gates and one memory unit. The three gates were the input gate, forget gate, and output gate, whose outputs could be represented as follows:

(5) $$i_{t}=\sigma \left(W_{i}x_{t}+U_{i}h_{t-1}+b_{i}\right),$$

(6) $$f_{t}=\sigma \left(W_{f}x_{t}+U_{f}h_{t-1}+b_{f}\right),$$

(7) $$o_{t}=\sigma \left(W_{o}x_{t}+U_{o}h_{t-1}+b_{0}\right),$$

where $x_{t}$ refers to the input, i.e., the taxi departure flow matrix in time slot t, $h_{t-1}$ is the output of the LSTM in the last time slot, W and U represent weights, b is the bias; $i_{t}$, $f_{t}$, and $o_{t}$ are the outputs of the input gate, forget gate, and output gate, respectively, in the current time slot, and σ() is a non-linear activation function.

The updates to the memory unit and output of the LSTM were determined with the following equations:

(8) $$c_{t}=f_{t}⊙c_{t-1}+i_{t}⊙\tanh\left(W_{c}x_{t}+U_{c}h_{t-1}\right),$$

(9) $$h_{t}=o_{t}⊙\tanh\left(c_{t}\right),$$

where W and U are weights, $c_{t}$ and $c_{t-1}$ are the state of memory units in the time slots t and t−1, respectively, $h_{t}$ is the output of the LSTM at the current time slot, ⊙ represents the Huffman product, and tanh() is a non-linear activation function. After processing this module, the dimensions of the graph were reduced to 1×R.

#### 5.2.3. Feature Fusion Module

After processing by the former two modules, two kinds of feature matrices were obtained: $O_{local}$ for the local road section relationship graph, and $O_{global}$ for the global road section relationship graph. The dimensions of the feature matrices were 1×R. To combine the two kinds of information, $O_{local}$ and $O_{global}$ were concatenated to form a new matrix $O_{pre}$ with the dimensions of 2×R. Then, a 2D convolution was performed on $O_{pre}$ using a convolution kernel whose size was 2×1. The stride was 1, and the padding parameter was set to VALID. The output, denoted as $X^{t+1}$, with the dimensions of 1×R, predicted the taxi departure or arrival flows in the next time slot t+1.

#### 5.2.4. Loss Function

In this research, we took the departure and arrival flow predictions as two related tasks, so the loss function was designed to reflect the impacts of these two kinds of predictions. The first part of the loss function was the departure flow prediction loss, $loss_{out}$, defined as

(10) $$loss_{out}={\left\|Y_{t\_out}-{\hat{Y}}_{t\_out}\right\|}_{2},$$

where ${\hat{Y}}_{t}$ is the true value of the departure flow in the road section and $Y_{t}$ is the predicted value.

The second part of the loss function, that is, $loss_{in}$, referred to the loss in arrival flow prediction, which was defined as

(11) $$loss_{in}={\left\|Y_{t\_in}-{\hat{Y}}_{t\_in}\right\|}_{2},$$

To avoid overfitting, an extra $L_{2}$ regularization item was added, so the total loss became

(12) $$loss=\lambda_{1}loss_{out}+\lambda_{2}loss_{in}+\lambda_{3}L_{2},$$

where $\lambda_{i}$, i=1,2,3 is a super-parameter.

## 6. Experiments

In this section, we describe how we verified the effectiveness of the proposed model and compared it to the existing methods. Finally, an ablation analysis was presented to show the performances of different modules.

### 6.1. Dataset

The dataset used in the experiments contained real-world taxi trajectories from the city of Xi’an, a large city in northwestern China. The trajectories came from more than 10,000 taxis, and they covered 20,000 road sections. In each taxi, a GPS device recorded the vehicle ID, time stamp, longitude and latitude, vehicle speed and driving direction, and the state code of the vehicle. A state code of “4” indicated that the vehicle was vacant, and a “5” indicated that the vehicle was carrying passengers. The state code changed from “4” to “5” to indicate that passengers had entered, and the state code changed from “5” to “4” to indicate that passengers had alighted. This kind of record was captured every 30 s. An example of the raw taxi trajectory data is shown in Table 1.

We used seven days of taxi trajectories generated during 17–23 October 2016, of which, six days of data were used as the training set and the remainder of the dates were used as the test set. In the experiments, we selected road sections whose departure flows were greater than 15 times per day, which resulted in 851 road sections being chosen. In the existing research, road sections in which departure flows are less than a certain time, such as 15 per day, are not regarded as main road sections; rather, this represents road sections such as a small branch linking to a main road. The influences of these road sections on the flow prediction are weak and often ignored in most research [10]. Likewise, these influences were not considered in this research. Furthermore, we divided each day into 288 time slots, with each time slot being five minutes long.

### 6.2. Evaluation Metrics

Two metrics were used to evaluate the performance of the prediction models: root-mean-square error (RMSE) and mean absolute error (MAE), defined as follows:

(13) $$RMSE=\sqrt{\frac{1}{N}{\sum_{i=1}^{N}\left(y_{i}-{\hat{y}}_{i}\right)}^{2}},$$

(14) $$MAE=\frac{1}{N}\sum_{i=1}^{N}\left|y_{i}-{\hat{y}}_{i}\right|,$$

where $y_{i}$ refers to the departure flow predicted in the $i^{th}$ road section, ${\overset{⌢}{y}}_{i}$ is the true value, and N is the total number of road sections.

### 6.3. Model Training

The experiments were conducted based on the TensorFlow framework, and an end-to-end approach was used in model training. In the experiment, the learning rate was set to 0.0001, the batch size was set to 32, the number of iterations was set to 500, and an early termination strategy was applied to avoid overfitting. The data from 17 October to 22 October 2016 were used for training, and the data for October 23 were used for testing. The departure flow sequence corresponding to the last two hours was used to predict the departure flow in the next time slot. We optimized the parameters to minimize the loss function defined in Equation (12), and the Adam (Adaptive Moment Estimation) gradient descent algorithm was used to train the network. In the experiment, a multitask learning strategy with parameter sharing was adopted. The spatial and temporal feature extraction modules shared layer parameters. The input of a single batch included the feature matrices of the departure and the arrival flow graphs for multitask learning.

In the local relationship graphs, the adjacency matrix was generated according to the connectivity of the road sections in the traffic road network. Hence, the maximum degree of the node was 3. In the global relationship graphs, the element in the adjacency matrix was the similarity between the departure flow sequence and the arrival flow sequence of the road sections. In the global relationship graph for departures, more than 60% of the nodes had a degree less than 50, and the same was true in the global relationship graph for arrivals.

The GPU deep learning workstation was used in the experiment. The workstation was equipped with two Intel(R) Xeon(R) E5-2690 CPUs, a memory of 256 GB and an RTX 2080Ti GPU accelerator cards for the deep network training and testing. The training time of this model was around one hour, and the running time of the prediction was around 500 milliseconds. This ensured real-time prediction of the departure flow.

**Figure 6:**
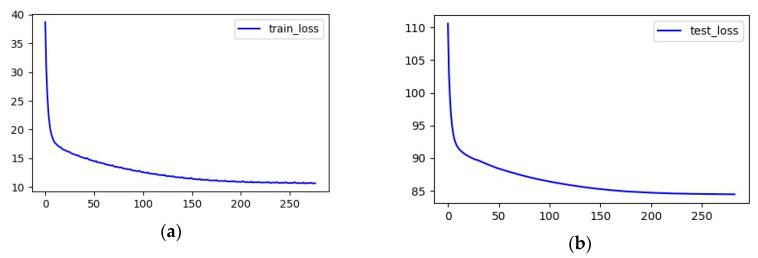
(**a**) The loss curve of the training set; (**b**) the loss curve of the testing set.

**Figure 7:**
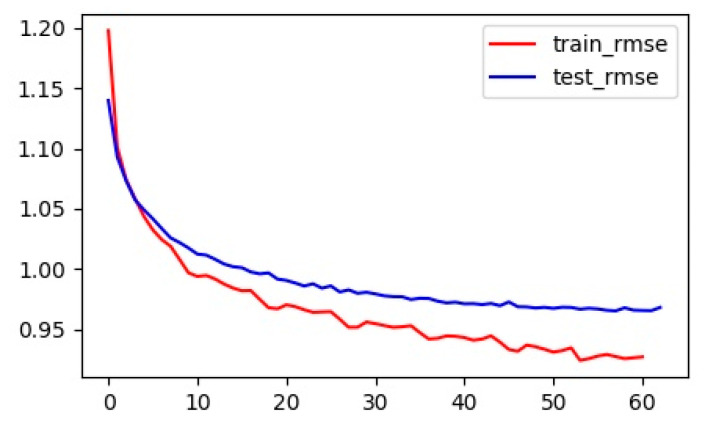
RMSE curve.

The loss function curve during the model training process is shown in Figure 6. It can be seen from the figure that our model was properly trained, and it was not overfitted. Figure 7 shows the RMSE curve during model training.

### 6.4. Comparison with Common Traffic Prediction Models

We compared the proposed prediction models with eight typical methods, which are described below:

- Historical average (HA). The average taxi demand values in historical time slots are used to estimate the future demands. In this research, the historical time slots were all the time slots in the training set corresponding to the same time slot to be predicted.
- Auto-regressive integrated moving average (ARIMA). ARIMA combines autoregressive, moving average, and difference methods to predict time series.
- Multiple layer perceptron (MLP). MLP is a common neural network prediction model. The MLP in this research contained three fully connected layers, which could predict the taxi demands in the next time slot by inputting a historical demand sequence.
- Support Vector Regression (SVR). SVR uses linear regression to predict the time series, mainly by constructing a linear decision function in a high-dimensional space after upgrading the dimensions.
- Long Short-Term Memory network (LSTM). LSTM is a recurrent neural network that can effectively capture the long-term correlation of time series and that is very suitable for time series prediction.
- Graph Convolutional Network (GCN). The GCN solves the problem of the CNN only being able to deal with two-dimensional regular grid data. It also extends the convolution operation to the graph domain, extracts complex node relationships in graph data, and has a strong ability to extract spatial correlation. In this research, the graph was constructed according to the global relationship of the road sections.
- GCN-LSTM (Multitask). The input data for GCN-LSTM includes a feature matrix and adjacency matrix for the above global relationship graph. First, the two-layer GCN + GLU is used to extract spatial features, then the LSTM network is used to extract temporal features, and the multitask learning strategy is applied in training. At the same time, the taxi departure and arrival flows can be predicted.
- T-GCN [26]. T-GCN integrates GCN and GRU. GCN is used to learn complex topological structures and capture spatial dependence, and GRU is used to learn the dynamic changes in traffic data to capture temporal dependence.

As shown in Table 2, the proposed MGLN prediction network obtained the lowest RMSE and MAE, mainly because our method account for both the local and global correlations between the taxi departure flows on the road sections. Our method also used the correlation between the departure and arrival flows to improve the prediction performance, and it fully explored the spatial and temporal characteristics. ARIMA, MLP, and SVR could not extract the long-term dependence of the data effectively. Although LSTM could extract the long-term dependence effectively, it could not mine its spatial characteristics. The GCN was unable to mine the temporal features. The shortcoming of GCN-LSTM (Multitask) was that it did not consider the local correlation between the departure flow of taxis on road sections. In T-GCN, GRU was used to extract temporal features. Compared with LSTM, GRU was simpler and it had lower training complexity, but its ability to extract long-term dependence was insufficient.

### 6.5. Ablation Analysis

In this section, we illustrate the effectiveness of MGLN modules through experiments. Table 3 shows the experimental error results in the validation of each module. The single-task GCN (Global) method was used as a baseline, and it only used the relationship similarity between the road departure flow sequences; that is, it only considered the global correlation of the road sections, and it did not use a multi-task learning strategy to predict the arrival flow at the same time. The network structure was a two-layer GCN, and the RMSE and MAE were 0.9879 and 0.6869, respectively.

1. GLU Unit

The Single-Task GCN + GLU (Global) method added the GLU unit to the single-task GCN (Global) method. With this method, both the RMSE and MAE decreased, which were 0.9842 and 0.6836, respectively, showing that the GLU unit could improve the prediction performance.

2. Multitask Learning

The Multitask GCN + GLU (Global) method considered the correlation between the departures and arrivals. For this method, the taxi arrival flow prediction was regarded as the related task, and a multitask learning strategy was used to help improve the accuracy of predicting taxi trip flows. It could be seen that this method further reduced the prediction error compared to the single-task GCN + GLU (Global) method, and the RMSE and MAE were 0.9774 and 0.6781, respectively.

3. Temporal feature extraction module

In the multitask GCN + GLU + LSTM (Global) method, the temporal feature extraction module was added. The prediction error range increased with this method compared with the above method. The RMSE and MAE were 0.9556 and 0.6688, respectively, indicating the importance of fully extracting time features in the prediction.

4. Effect of the spatial local features on the departure flow prediction

The multitask GCN + GLU + LSTM (Local) method only used the connected relation composition between the road sections in the original road network to predict the departure flow. The obtained RMSE and MAE were close to those of the multitask GCN + GLU + LSTM (Global) method, indicating that spatial local characteristics also played an important role in predicting the travel flow.

Our method considered all of the above factors and had a minimum prediction error.

### 6.6. Performance of the Model with Different Time Slot Lengths

**Figure 8:**
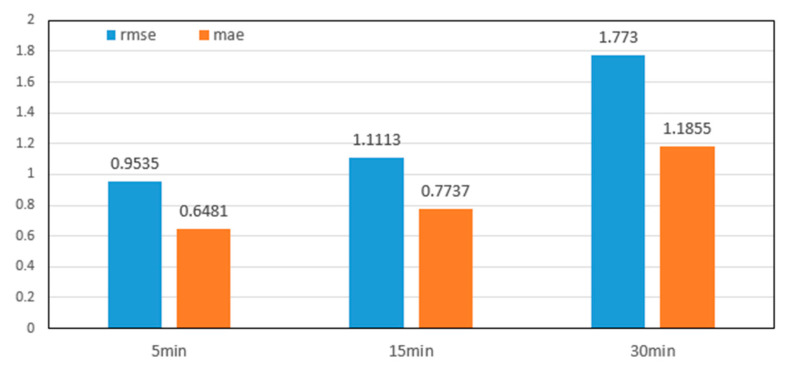
Impacts of the length of the time slot on the prediction performance.

As described in this section, we divided the time slots into different lengths. We used the MGLN Prediction Network to predict the travel flow to verify the stability of the network. As shown in Figure 8, the time slot lengths we adopted were 5, 15, and 30 min. As the time slot length increased, the prediction error also increased by a small range, which indicated that the proposed method was more applicable to predict taxi demands on a smaller scale in terms of the time granularity.

### 6.7. Performance of the Model with Different Lengths of the Prediction Time Step

As described in this section, we predicted $X^{k+1}$, $X^{k+2}$, …, $X^{k+5}$ using our model. We could see the impacts of the length of the prediction time step on the model performance, as shown in Table 4. As the length of prediction time step increased, both the RMSE and the MAE increased because the prediction errors accumulated easily with the steps in the multistep prediction. Each predicted value was fed back to the input as a new input. As the window slid, the actual data were reduced, the prediction data increased, and the error accumulated and was amplified, thereby affecting the long-term prediction ability of the algorithm. This was the biggest problem of the multistep prediction.

## 7. Conclusions

In this research, we designed a taxi departure flow prediction network that was able provide predictions for a finer spatial granularity, that is, the road section level. The network took local and global correlations between the road sections in the road network into consideration, fully exploited these two spatial correlations based on the graph convolutional neural network, and was able to fully extract temporal features based on the LSTM network. In addition, we used a multitask learning strategy, taking the taxi arrival flow prediction as the relevant task, to help improve the accuracy of the departure flow prediction. As the external environmental factors such as weather, wind speed, and season also affect the taxi travel flow, such extraneous factors will be considered in our future work to try to further reduce prediction errors. Additionally, in future work, we will try to optimize the network structure to achieve a more accurate taxi travel flow prediction, including long-term and multistep prediction.

## Figures and Tables

**Table 1:** Example of Raw Taxi Trajectory Data.

Vehicle ID	Timestamp	Longitude	Latitude	Speed	Direction	State Code
XXXXXX	2016-10-20 00:13:30	108.920168	XXX	0	0	4

**Table 2:** Model Comparison

Method	RMSE	MAE
HA	1.0126	0.6924
ARIMA	1.2746	1.1289
MLP	1.1837	0.7228
SVR	1.0088	0.6916
LSTM	1.0048	0.6831
GCN	0.9879	0.6769
GCN-LSTM(Multi-Task)	0.9556	0.6688
T-GCN	0.9832	0.6728
MGLN	0.9535	0.6481

**Table 3:** Results of ablation analysis

Method	RMSE	MAE
Single-task GCN (Global)	0.9879	0.6869
Single-task GCN + GLU (Global)	0.9842	0.6836
Multitask GCN + GLU(Global)	0.9774	0.6781
Multitask GCN + GLU + LSTM (Global)	0.9556	0.6688
Multitask GCN + GLU + LSTM(Local)	0.9544	0.6643
MGLN	0.9535	0.6481

**Table 4:** Impacts of the length of the prediction time step on the prediction performance.

Length of Prediction Time Step	RMSE	MAE
1	0.9535	0.6481
2	1.0582	0.7709
3	1.0604	0.7715
4	1.0678	0.7803
5	1.0701	0.7852
